# A Three lncRNA Set: AC009975.1, POTEH-AS1 and AL390243.1 as Nodal Efficacy Biomarker of Neoadjuvant Therapy for HER-2 Positive Breast Cancer

**DOI:** 10.3389/fonc.2021.779140

**Published:** 2021-12-06

**Authors:** Zhao Bi, Peng-Fei Qiu, Yue Zhang, Xing-Guo Song, Peng Chen, Li Xie, Yong-Sheng Wang, Xian-Rang Song

**Affiliations:** Shandong Cancer Hospital and Institute, Shandong First Medical University and Shandong Academy of Medical Sciences, Jinan, China

**Keywords:** breast cancer, neoadjuvant therapy, HER-2, LncRNA-AC009975.1, POTEH-AS1, lncRNA-AL390243.1, biomarkers

## Abstract

**Purpose:**

The study aimed to explore whether the expression of lncRNAs in primary tumors could predict nodal efficacy after neoadjuvant therapy (NAT) for HER2+ breast cancer.

**Methods:**

Total RNA was extracted from HER2+ breast cancer tissues before NAT (n=103) and from 48 pairs of cancers and para-cancers tissues that did not receive NAT. Different lncRNAs were selected by microarray, validated by qPCR, and analyzed to illuminate their potential as nodal efficacy biomarkers after NAT.

**Results:**

Our results demonstrated that three lncRNA sets, lncRNA-AL390243.1, POTEH-AS1, and lncRNA-AC009975.1, were up-regulated in non-apCR tissues. The AUC value was 0.789 (95%CI: 0.703-0.876). The multivariate logistic regression analysis identified the expression of lncRNA-AL390243.1 (OR 5.143; 95% CI: 1.570-16.847), tumor type (OR 0.144; 95% CI: 0.024-0.855), and nodal stage (OR 0.507; 95% CI: 0.289-0.888) as independent predictors for apCR after NAT in HER2+ patients (all *p*<0.05). Then the three predictors were used to create a predictive nomogram. The AUC value was 0.859 (95%CI: 0.790-0.929). The calibration curve showed a satisfactory fit between predictive and actual observation based on internal validation with a bootstrap resampling frequency of 1000. Patients with higher expression of lncRNA-AL390243.1 had worse survival. LncRNA-AL390243.1 was up-regulated more in the nodal positive subgroup than in the nodal negative subgroup (*p*=0.0271).

**Conclusion:**

The lncRNA-AL390243.1, POTEH-AS1, and lncRNA-AC009975.1 were upregulated in non-apCR breast cancer tissues. These three lncRNAs might have the potential to be used as predictive biomarkers of nodal efficacy of HER2+ breast cancer. Further studies are required to illuminate the underlying molecular mechanisms further.

## Introduction

Neoadjuvant therapy (NAT) has become an adapt therapy for patients with inoperable as well as some high-risk breast cancer, such as stage II–III HER-2 positive (HER2+) and triple-negative breast cancer (TNBC) (1, 2). A major clinical benefit of NAT is the downstaging of the tumor. As a result, patients with clinical nodal positive (cN_+_) disease might have more chance of achieving axillary nodal pathological complete response (apCR) and omit axillary lymph node dissection (ALND) after NAT (3). At the same time, identification of apCR and subsequent omission of ALND could prevent the short- and long-term side effects of ALND such as lymph edema, limited range of motion of the shoulder, and numbness of the upper arm (4). In the era of sentinel lymph node biopsy (SLNB), the feasibility of SLNB has been confirmed in selected cases (category 2B) for patients with initial cN_+_ and ycN_0_ disease after NAT (5, 6). The result of the Z1071 trial showed that for patients with initial cN_+_ and ycN_0_ disease after NAT, using combined tracers (radiolabeled colloid and blue dye) and detecting > 2 negative sentinel lymph nodes (SLN), the false negative rate (FNR) of SLNB after NAT could reduce to 10% or less (7). In the 2021 St. Gallen International Breast Cancer Consensus Conference, the panel recommended that patients with initial cN_1_ disease and convert to ycN_0_ disease after NAT, are potential candidates for SLNB (8). However, ALND remains the standard option in axillary management since the overall false negative rate (FNR) of SLNB after NAT is still a concern. Given these concerns, optimizing patient selection might be necessary to support the use of SLNB as an alternative to ALND after NAT (9).

The apCR rates after NAT for hormone receptor positive/HER-2 negative (HR+/HER2-), HER2+ and TNBC subtype were 17.0-21.2%, 51.2-82.1% and 41.0-67.0%, respectively (1). Compared with other subtypes of breast cancer, HER2+ patients might have more chances of achieving apCR. Predicting apCR after NAT of HER2+ patients has clinical implications.

LncRNA is a category of RNA with over 200 nucleotides that do not have protein-coding ability and are characterized by low sequence conservation and complexity in regulatory mechanism (10). Recently, lncRNA has gained more and more attention from researchers, and their roles in a variety of cancers, especially in breast cancer have also been gradually revealed. A large number of studies have demonstrated the potential value of lncRNA as efficacy prediction biomarkers for breast cancer (11–13). However, most studies focused on the efficacy prediction of breast primary tumors. To our knowledge, up to now, fewer studies have explored whether lncRNA was associated with nodal efficacy after NAT. At the same time, the breast pathological complete response (bpCR) after NAT was different from the apCR. This difference was most significant in the HER2+ subgroup (**Table 1**). Therefore, it was not comprehensive to use efficacy biomarkers of the primary tumor to predict the nodal efficacy after NAT.

**Table 1:** The apCR and bpCR after neoadjuvant in different molecular subtypes.

	overall		HER2+ subgroup		HR+/HER2-subgroup		TNBC subgroup	
	apCR	non-apCR	apCR	non-apCR	apCR	non-apCR	apCR	non-apCR
bpCR	78	40	42	22	16	11	20	7
non-bpCR	77	221	39	69	28	130	10	22

apCR, axillary nodal pathological complete response; bpCR, breast pathological complete response.

The purpose was to explore whether the expression of lncRNA in primary tumors could predict nodal efficacy after NAT for HER2+ breast cancer. In the current study, we first screened the differential lncRNAs between the apCR group and non-apCR group in the HER2+ subgroup using microarray and then validated them in a larger cohort *via* Reverse Transcription and quantitative PCR (RT-qPCR). Then we analyzed their diagnostic efficiency, thus revealing the crucial role of lncRNA as a nodal efficacy biomarker after NAT for HER2+ breast cancer.

## Methods and Materials

### Patients

The medical records of breast cancer patients who had histology confirmed invasive ductal carcinoma and initial clinical staging T_1-4_N_1-3_M_0_ treated in Shandong Cancer Hospital and Institute were retrospectively reviewed to analyze the consistency of apCR and bpCR.

A total of 103 core biopsy fresh tissue samples (from April 2014 to 2019) were taken from HER2+ patients who received NAT. These patients received a standard dose of four cycles of anthracycline and cyclophosphamide followed by four cycles of taxane every 3 weeks prior to surgery. All patients received anti-HER2 target therapy. At the same time, 48 pairs of fresh tissue samples (from July 2020 to December 2020) were also taken from HER2+ patients who did not receive NAT. Patients were ineligible if they had undergone therapy prior to NAT, concurrent cancer, bilateral breast cancer, or distant metastases.

The study was approved by the Shandong Cancer Hospital Ethics Committee (No. SDTHEC20110324). Informed consent was obtained from all patients, and all procedures were in accordance with the ethical standards of the responsible institutional committee on human experimentation and with the Helsinki Declaration.

### Treatment

The clinical stage was determined in accordance with the eighth TNM staging system by the American Joint Committee on Cancer. All clinicopathological factors and laboratory indexes, including age, clinical tumor stage, clinical nodal stage, tumor type, molecular subtypes, neutrophil count, lymphocyte count, monocyte count, plasma blood sugar, platelet count, hemoglobin, plasma fibrinogen, and D-dimer, were estimated before the NAT and were collected from the patients’ medical record.

Before NAT, core biopsies of the breast tumor were taken, guided by ultrasound. Following core biopsies, excised tissue specimens were immediately placed in liquid nitrogen and subsequently frozen at −80°C. Suspicious positive axillary lymph node (ALN) was also accessed by fine-needle aspiration prior to initiation of NAT. We detected image positive lymph nodes by ultrasonography. Morphologic characteristics predictive of imaging positive lymph nodes detected by ultrasound had a cortical thickness greater than 2.5-3.0 mm, focal cortical lobulation, loss of the fatty hilum, a round shape, and abnormal cortical blood flow. Positive HR status was defined as at least one percent of tumor cells expressing the receptor. HER-2 status was determined based on the American Society of Clinical Oncology/College of American Pathologists guidelines. Positive HER-2 was defined as HER-2 overexpression (3+) detected by immune-histochemical staining or fluorescence *in situ* hybridization (14). After this evaluation, patients received a standard dose of four cycles of anthracycline and cyclophosphamide followed by four cycles of taxane every 3 weeks prior to surgery as the preferred regimens among the available treatments. HER2+ patients received anti-HER2 target therapy: trastuzumab and (or) pertuzumab.

After finishing the system therapy, all patients received full ALND with at least ten nodes from anatomical levels I-III. Local treatment of the breast included breast-conserving surgery and mastectomy. According to the pathological assessment, these specimens were divided into four groups: ypT_0_N_0_, ypT_+_N_+_, ypT_0_N_+,_ and ypT_+_N_0_. The apCR was defined as no residual carcinoma in the axilla (ypN_0_). The bpCR was defined as no residual invasive carcinoma in the breast.

### RNA Isolation

Tissue samples were cut into small pieces with a weight of 40-50 mg, then thoroughly ground in a dedicated mortar and transferred into a 1.5 mL centrifuge tube. Then 1 ml Trizol reagent (Thermo Fisher Scientific, Waltham, MA, USA) was added to each centrifuge tube to extract RNA according to the procedure.

### LncRNA Microarray

The Arraystar human lncRNA microarray V5.0 was designed for the global profiling of human lncRNA. About 19307 lncRNAs can be detected by third-generation lncRNA microarray. Arraystar maintains high quality proprietary lncRNA transcriptome databases that extensively collect lncRNA through all major public databases and scientific publications.

Briefly, a total of 2μg RNA from each sample isolated with the RNeasy Mini kit (Qiagen, Hilden, Germany) was labeled with the Agilent Gene Expression Hybridization Kit (Agilent), after which, the slides were scanned with the Agilent Microarray Scanner (Agilent). A mixture of equal amounts of total RNAs from each group was used as the reference pool. The Feature Extraction software (version 10.7.1.1, Agilent) was used to analyze array images to get raw data and Genespring software (version 14.8, Agilent) was employed to finish the basic analysis with the raw data. R language was utilized for analyzing the differentially expressed lncRNA.

### RT-qPCR

The 5μg isolated RNA was reverse-transcribed into complementary DNA (cDNA) using Takara PrimeScript RT reagent Kit (Takara Bio, Kusatsu, Japan) in a 10μl reaction system according to the manufacturer’s instructions. Furthermore, LightCycler 480 qPCR system (Roche Diagnostics, BALE, Germany) was used for qPCR with a 20μl reaction system, including 10μl of SYB-Green Premix Ex Taq II Reagent (TakaRa Bio, Nojihigashi, Kusatsu, Japan), 7.2μl of RNase-free water, 0.4μl of upstream and 0.4μl of downstream primers, and 2μl of cDNA template. The reactions started at 95°C for 10 min, followed by 45 cycles of 95°C for 15 s, 60°C for 1 min. The qPCR primers are listed in **Table 2**. All experiments were carried out in duplicate, and then the median Ct value was calculated, GAPDH was used as an internal reference gene. The relative expression of lncRNA was evaluated by the comparative cycle threshold (ΔCt) method: (ΔCt = Ct_lncRNA_–Ct_GAPDH_) as described previously (15).

**Table 2:** The qPCR primers.

Gene	Forward primer	Reverse primer
lncRNA-AC009975.1	CAGTGCTGCCATCCCTAGTTGC	AACTCCAAGAGGTGGTTGCATAGC
POTEH-AS1	TTATGGATATGTGCCGCCGAAGC	GGACTCAACAGAGCCAAGCCTTG
lncRNA-AL390243.1	GCTGCCTGCTGATGCCATCG	TCCACTGCTGAGAGTCCTGTAAGG
GAPDH	CCTCG CCTTT GCCGA TCC	GGATC TTCAT GAGGT AGTCA GTC

### Statistical Analysis

Statistical analyses were carried out using SPSS Statistics 22.0 software (IBM Corporation, Armonk, NY, USA) or GraphPad Prism version 9.0 (GraphPad Software, San Diego, CA, USA). Kolmogorov-Smirnov test was performed to verify whether the data were normally distributed or not. If the data followed normal analysis, unpaired t-test would be used; if not, Mann-Whitney test would be used. In paired data, the normally distributed numeric variables were evaluated by paired t-test, whereas non-normally distributed variables were analyzed by Wilcoxon rank-test. Univariable comparisons were performed using the Kruskal-Wallis test for continuous variables and Pearson χ^2^ test or Fisher exact test for categorical variables. Multivariable analyses were conducted using logistic regression models.

A nomogram was developed based on variables in the final model with *p*<0.05 using the “rms” package for R. Calibration of the nomogram was carried out by internal validation using the bootstrap resampling approach and was displayed using a calibration curve. The receiver operator characteristic (ROC) analysis was used to evaluate the diagnostic efficiency. Data were shown as mean ± Standard Deviation, a *p*-value < 0.05 was considered statistically significant, and all tests were set as double-tailed.

## Results

### The Consistency of apCR and bpCR

We first explored the consistency of apCR and bpCR in 416 patients. All patients received a standard dose of four cycles of anthracycline and cyclophosphamide followed by four cycles of taxane every 3 weeks prior to surgery from April 2014 to 2019. HER2+ patients received anti-HER2 target therapy. The apCR and bpCR rate was 37.3% (155/416) and 28.4% (118/416), respectively. The consistency coefficient of apCR and bpCR was 0.719 (*p*<0.001), and 0.789, 0.746, and 0.645 among the HR+/HER2-, TN, and HER2+ subtypes, respectively (**Table 1**).

### Identification of Differentially Expressed lncRNA Using Microarray in HER2+ Subtype

**Figure 1:**
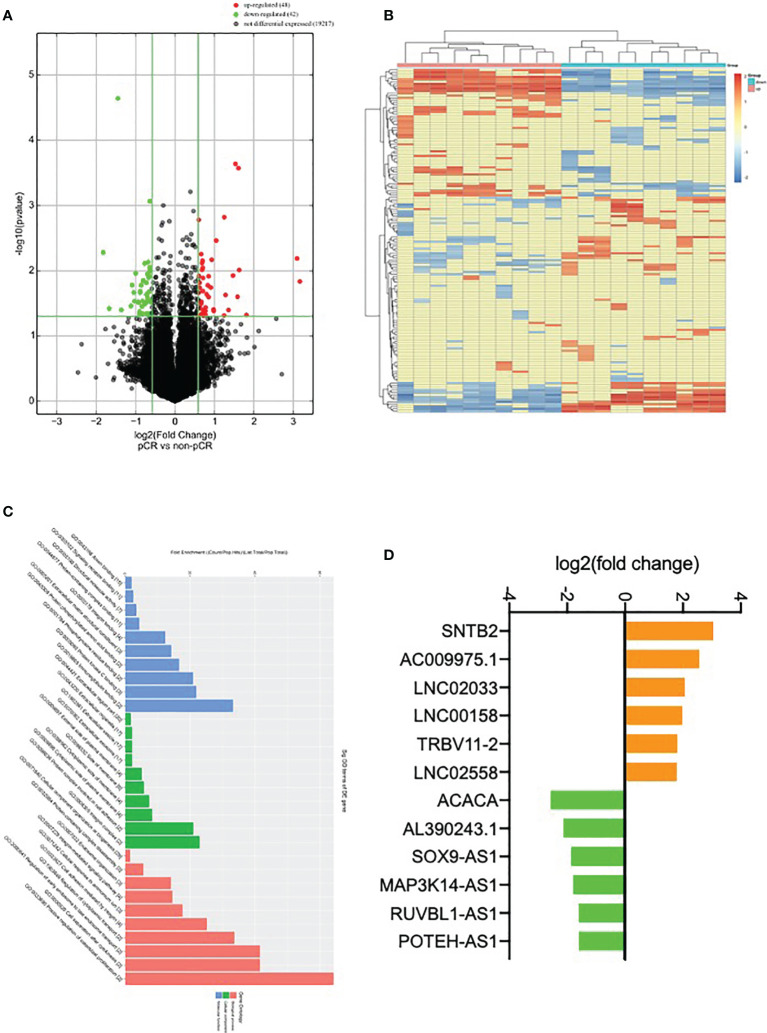
Identification of differentially expressed lncRNA. **(A)** Volcano plot compared the expression fold change of lncRNA for ypT_0_N_0_ and ypT_+_N_+_ tissues; **(B)** a heat map was generated after supervised hierarchical cluster analysis. LncRNA is shown in red (upregulation) versus blue (downregulation); **(C)** GO analysis of lncRNA in ypT_0_N_0_ and ypT_+_N_+_ tissues; **(D)** 12 selected lncRNAs are shown in yellow (upregulation) versus green (downregulation).

To explore the differentially expressed lncRNA, six HER2+ fresh core biopsy tissues before NAT were collected (3 tissues from ypT_0_N_0_ group and 3 tissues from ypT_+_N_+_ group) and subjected to lncRNA microarray. A total of 48 up-regulated genes and 42 down-regulated genes in ypT_0_N_0_ cases were screened and drawn in a volcano map, as shown in **Figure 1A**. The expression patterns of these differentially expressed lncRNA were shown as the heatmap using hierarchical cluster analysis (**Figure 1B**), and the Gene Ontology functional classification was performed to gain an overall insight into the functions of annotation genes (**Figure 1C** **)**. Finally, six up-regulated and six down-regulated lncRNAs with significance were selected as the candidates for the next validation (**Figure 1D**).

### The lncRNA-AL390243.1, POTEH-AS1, and lncRNA-AC009975.1 Were Associated With apCR

One hundred and three HER2+ patients received a standard dose of four cycles of anthracycline and cyclophosphamide followed by four cycles of taxane every 3 weeks plus trastuzumab and (or) pertuzumab were enrolled in the qPT-PCR cohort. The apCR and bpCR rate was 59.6% (62/103) and 38.8% (40/103) in 103 HER2+ patients, respectively. Among the 78 patients who received trastuzumab, the apCR and bpCR rate was 56.4% (44/78) and 33.3% (26/78), respectively. Among the 25 patients who received trastuzumab and pertuzumab, the apCR and bpCR rate was 72.0% (18/25) and 56.0% (14/25), respectively.

**Figure 2:**
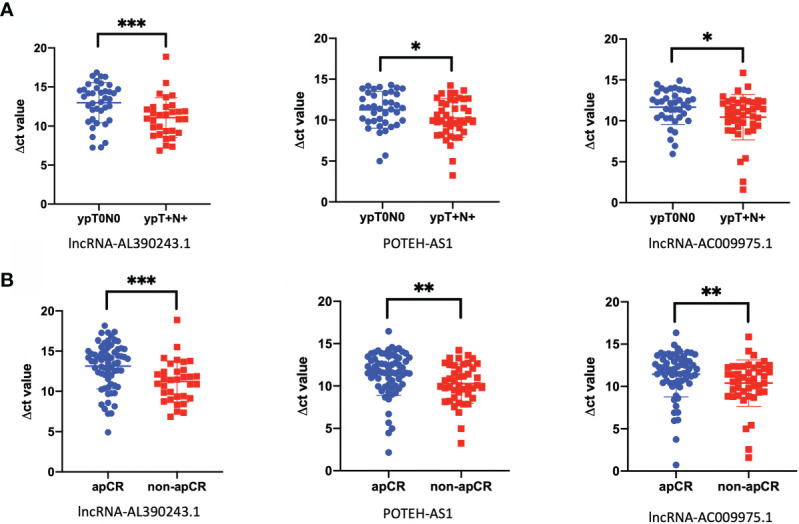
The lncRNA was closely related to nodal efficacy. **(A)** The different expression of lncRNA-AL390243.1, POTEH-AS1, and lncRNA-AC009975.1 in tissues in 39 cases of ypT0N0 group compared with 31 cases of ypT+N+ group. **(B)** The different expressions of lncRNA-AL390243.1, POTEH-AS1, and lncRNA AC009975.1 in tissues in 62 cases from the apCR group compared with 41 cases in the non-apCR group. *p < 0.05; **p < 0.005; ***p < 0.0005.

We then analyzed the differential expression of the above selected lncRNA expression by RT-qPCR in the HER2+ cohort, with 39 cases of the ypT_0_N_0_ group and 31 cases of the ypT_+_N_+_ group. After Mann-Whitney tests analysis, the expression of lncRNA-AL390243.1, POTEH-AS1, and lncRNA-AC009975.1 were elevated significantly in the ypT_0_N_0_ group compared with the ypT_+_N_+_ group (*p*=0.0008, *p*=0.0339, and *p*=0.0230, respectively, **Figure 2A**). Then, we analyzed the expression of these three lncRNA in the apCR (n=62) and non-apCR (n=41) groups. As shown in **Figure 2B**, the RT-qPCR result showed that the expression of lncRNA-AL390243.1, POTEH-AS1, and lncRNA-AC009975.1 was elevated significantly in the non-apCR group compared with the apCR group (*p*=0.0003, *p*=0.0022, and *p*=0.0090, respectively). Therefore, we selected lncRNA-AL390243.1, POTEH-AS1, and lncRNA-AC009975.1 as potential biomarkers of nodal efficacy.

**Figure 3:**
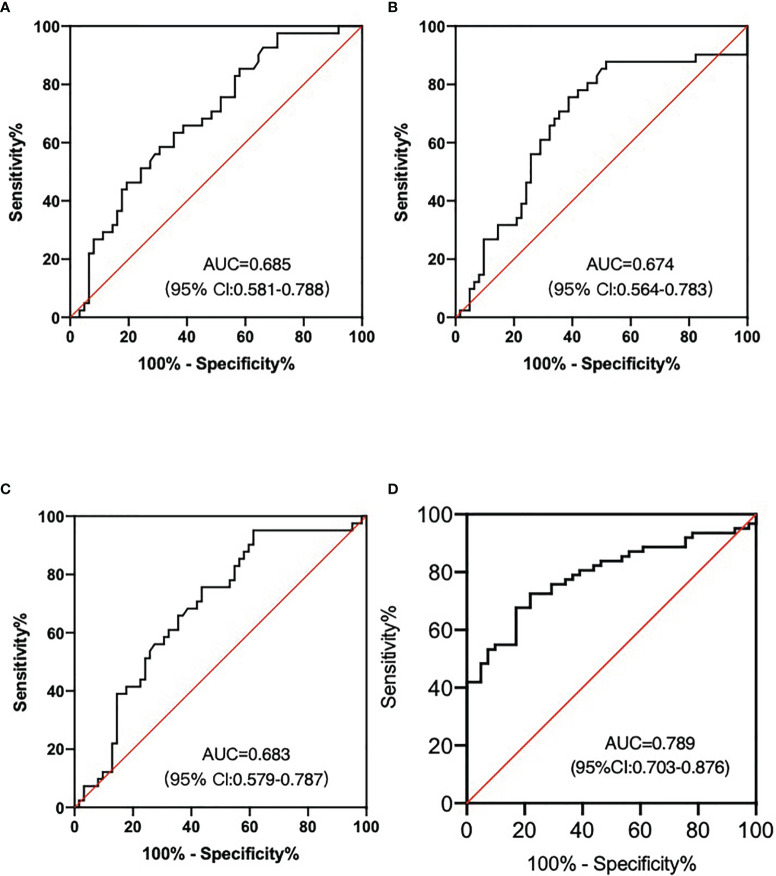
LncRNA as the nodal efficacy biomarkers after NAT for HER2+ breast patients. **(A)** The AUC of lncRNA-AL390243.1 was 0.685. **(B)** The AUC of POTEH-AS1 was 0.674. **(C)** The AUC of lncRNA-AC009975.1 was 0.683. **(D)** The diagnostic performance for their combination demonstrated the AUC of 0.789.

Then we evaluated the diagnostic efficiency of these three lncRNA in predicting nodal efficacy. The AUC value of lncRNA-AL390243.1, POTEH-AS1, and lncRNA-AC009975.1 was 0.685 (95%CI: 0.581-0.790) with 75.8% sensitivity and 46.1% specificity (**Figure 3A**), 0.674 (95%CI: 0.564-0.783) with 69.4% sensitivity and 58.5% specificity (**Figure 3B**), 0.683 (95%CI: 0.579-0.787) with 72.6% sensitivity and 56.1% specificity (**Figure 3C**), respectively (all *p*<0.05). When combined, the AUC value of the three lncRNA set reached 0.789 (95%CI: 0.703-0.876) with 80.6% sensitivity and 61.0% specificity (**Figure 3D**).

The expression of lncRNA and clinical characteristics is shown in **Table 3**. The expression of lncRNA AL390243.1 was irrelevant for age, HR status, tumor stage, nodal stage, and tumor type. The expression of POTEH-AS1 was correlated with progesterone receptor change, but irrelevant for other characteristics. The lncRNA-AC009975.1 was associated with tumor stage and nodal stage, but irrelevant with other characteristics.

**Table 3:** The relationship between lncRNA expression level and clinical characteristics.

Characteristics	Cases	lncRNA-AL390243.1 *p* value	POTEH-AS1 *p* value	lncRNA-AC00975.1 *p* value
T stage		0.401	0.252	0.049
T_1_	8			
T_2_	56			
T_3_	19			
T_4_	20			
ER status		0.073	0.129	0.466
positive	59			
negative	44			
ER change		0.899	0.632	0.585
yes	13			
no	52			
PR status		0.755	0.716	0.882
positive	37			
negative	66			
PR change		0.162	0.037	0.240
yes	6			
no	59			
HER-2 change		0.785	0.403	0.907
yes	3			
no	63			
N stage		0.204	0.110	0.012
N_1_	48			
N_2_	23			
N_3_	32			
Tumor type		0.087	0.734	0.405
IDC 1	3			
IDC 2	94			
IDC 3	3			
others	3			

Taken together, these data suggested that the three lncRNA set might be a promising biomarker for nodal efficacy prediction after NAT of HER2+ breast cancer. Next, we want to combine clinical factors and genomics to analyze the influence factors of apCR.

### The Analysis of apCR Influence Factors

According to the ROC analysis, the optimal cut-off values of apCR influence factors were 0.40mg/L for D-dimer level, 2.96g/L for fibrinogen level, 5.95mmol/L for blood sugar, 206.5×10^9^/L for platelet, 141.5 g/L for hemoglobin, 4.413 for NLR, 13.02 for lncRNA-AL390243.1 expression, 11.225 for POTEH-AS1 expression, and 12.57 for lncRNA-AC009975.1 expression, respectively. The apCR rate after NAT was 60.2% (62/103). Combined with clinical factors and genomics, the apCR was associated with the expression of lncRNA-AL390243.1, POTEH-AS1, and lncRNA-AC009975.1, tumor type, and nodal stage (all *p*<0.05). Furthermore, according to the multivariate analysis result, the expression of lncRNA-AL390243.1 (OR 5.143; 95% CI: 1.570-16.847, *p*=0.007), tumor type (OR 0.144; 95% CI: 0.024-0.855, *p*=0.033), and nodal stage (OR 0.507; 95% CI: 0.289-0.888, *p*=0.018) were indicated as independent predictors for apCR after NAT in HER2+ patients **(** **Table 4** **)**.

**Table 4:** The logistic regression analyses of apCR after NAT.

Factors	apCR	non-apCR	Univariable *p* value	Multivariable *p* value
Tumor stage			0.485	
T_1_	6	2		
T_2_	36	20		
T_3_	10	9		
T_4_	10	10		
Nodal stage			0.005	OR 0.507; 95% CI: 0.289-0.888 *p*=0.018
N_1_	37	11		
N_2_	13	10		
N_3_	12	20		
Tumor type			0.022	OR 0.144; 95% CI: 0.024-0.855 *p*=0.033
IDC 1	2	1		
IDC 2	60	34		
IDC 3	0	3		
others	0	3		
ER status			0.066	
positive	31	13		
negative	31	28		
ER change				
yes	25	27	0.103	
no	3	10		
PR status				
positive	38	28	0.468	
negative	24	13		
PR change				
yes	25	34	0.719	
no	3	3		
HER-2 change				
yes	27	36	0.417	
no	2	1		
POTEH-AS1 level			0.003	0.074
<11.225	42	38		
≥11.225	20	3		
AC00975.1 level			0.011	0.362
<12.57	33	32		
≥12.57	29	9		
AL390243.1 level			<0.001	OR 5.143; 95% CI: 1.570-16.847 *p*=0.007
<13.02	24	31		
≥13.02	38	10		
TRBV level			0.092	
<12.15	20	20		
≥12.15	42	21		
RUVBL1-AS1			0.066	
<13.34	31	28		
≥13.34	31	13		
D-dimer			0.180	
<0.4 mg/L	31	15		
≥0.4 mg/L	31	26		
Fibrinogen level			0.547	
<2.96 g/L	34	20		
≥2.96 g/L	28	21		
NLR			0.494	
<4.413	29	22		
≥4.413	33	19		

**Figure 4:**
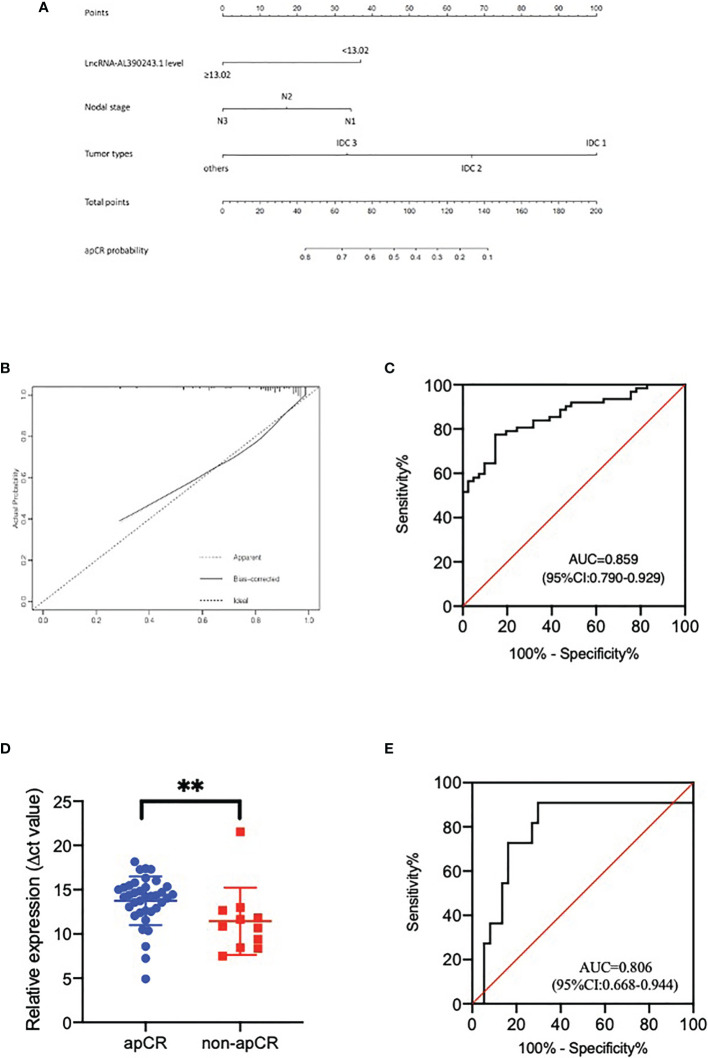
**(A)** The nomogram to predict patients with apCR. To calculate the probability of apCR, the scores for the three factors were summed up. And the total scores and bottom risk scale were referenced. **(B)** The calibration curve showed a satisfactory fit between the predictive and actual observation. **(C)** The ROC curve of the nomogram. **(D)** The different expression of lncRNA-AL390243.1 in tissues in the apCR group and non-apCR group among patients with cN_1_ disease. **p < 0.005. **(E)** The AUC of lncRNA-AL390243.1 was 0.806 among patients with cN_1_ disease.

Based on data obtained from the multivariate analysis, a nomogram was created to predict the probability of apCR after NAT (**Figure 4A**). To calculate the probability of apCR, the scores for these three factors were summarised. The total scores and bottom risk scale were referenced. The overall performance and discriminative performance of the model were assessed by the calibration curve and ROC curve analysis, respectively. Based on internal validation with a bootstrap resampling frequency of 1000, the calibration curve showed a satisfactory fit between the predictive and actual observation (**Figure 4B**). The ROC curve of the nomogram was depicted in **Figure 4C**. The AUC value was 0.859 (95%CI: 0.790-0.929, *p* < 0.001), indicating that the nomogram had a good discriminatory capability. These results indicated the great potential of the expression of lncRNA-AL390243.1, tumor type, and nodal stage as nodal efficacy biomarkers for HER2+ breast cancer. Integrating clinical factors and genomics might help to predict apCR and guide individualized treatment options.

As the 2021 St Gallen International Consensus Conference recommended that patients who present with cN_1_ before NAT and ycN_0_ disease after NAT are potential candidates for SLNB after NAT (8), we performed a subgroup analysis of patients with cN_1_ disease. The result of RT-qPCR showed that lncRNA-AL390243.1 was elevated significantly in the non-apCR group compared with the apCR group in the cN_1_ subgroup (*p*=0.0041, **Figure 4D**). The AUC value was 0.806 (OR 0.668, 95%CI= 0.668-0.944, *p*=0.002, **Figure 4E**). Multivariate analysis showed that lncRNA-AL390243.1 was indicated as independent predictors of apCR after NAT for the cN_1_ subgroup (*p*=0.012).

### LncRNA-AL390243.1 Facilitate Monitoring Survival After NAT

**Figure 5:**
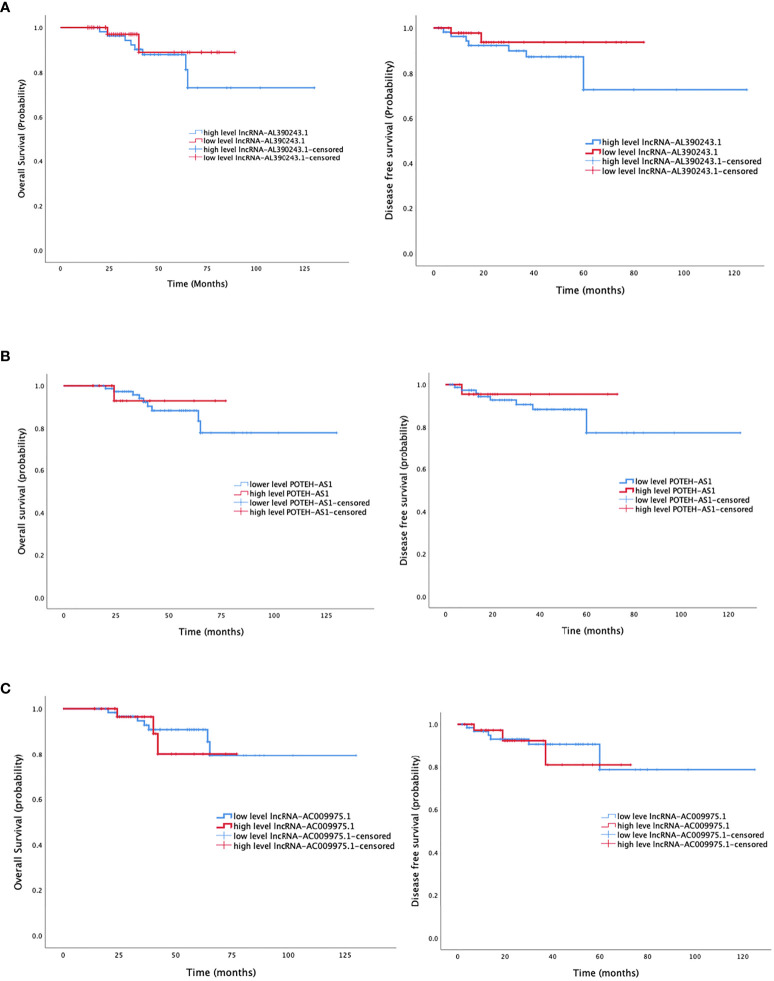
The survival analysis of three lncRNA. **(A)** The overall survival and disease-free survival of lncRNA-AL390243.1. **(B)** The overall survival and disease-free survival of POTEH-AS1. **(C)** The overall survival and disease-free survival of lncRNA-AC009975.1.

The median follow-up was 55 months (33-81 months), with the last follow-up in August 2021. Six cases were lost to follow-up. The median overall survival (OS) in patients with a low and high level of lncRNA-AL390243.1 expression was 50.0 and 30.5 months, respectively. The median disease-free survival (DFS) was 43.0 and 18.5 months, respectively in patients with a low and high level of lncRNA-AL390243.1 expression (**Figure 5A**). Patients with higher expression of lncRNA-AL390243.1 had worse survival compared to patients with a lower expression of lncRNA-AL390243.1. However, the expression of POTEH-AS1 and lncRNA-AC009975.1 was not associated with OS or DFS (all *p >*0.05, **Figures 5B, C**).

### The lncRNA-AL390243.1 Was Associated With Nodal Metastasis of Breast Cancer

**Figure 6:**
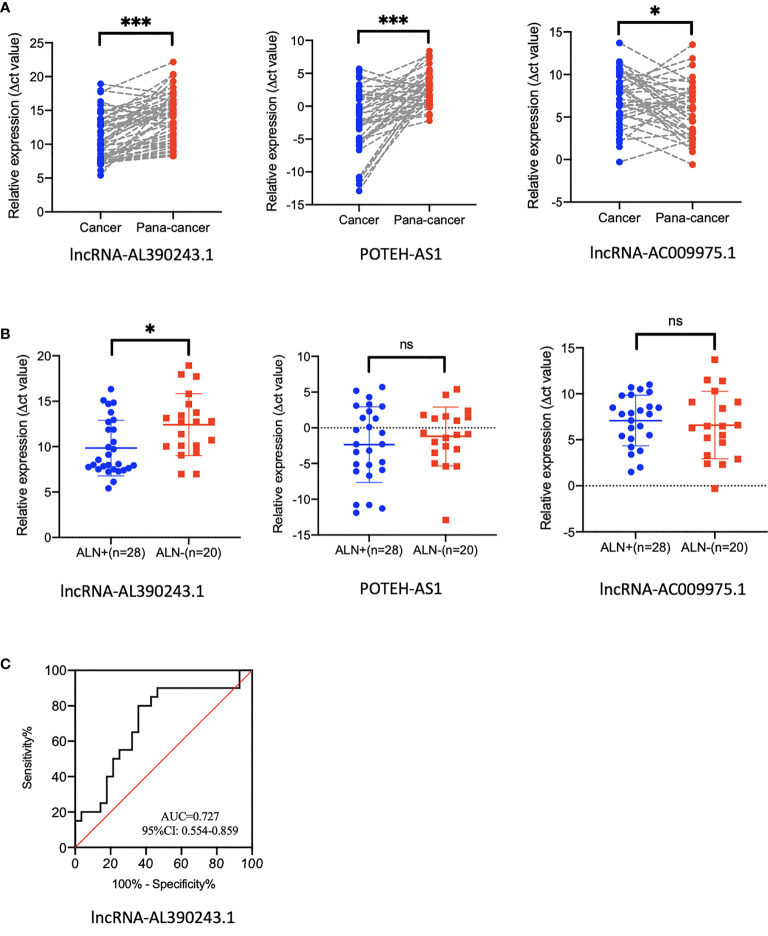
The expression of lncRNA in tissues that did not receive NAT. **(A)** The expression of lncRNA-AL390243.1, POTEH-AS1, and lncRNA-AC009975.1 in breast cancer tissue and para-cancer tissues. **(B)** The expression of lncRNA-AL390243.1, POTEH-AS1, and lncRNA-AC009975.1 in nodal positive tissues and nodal negative tissues. **(C)** The AUC value of lncRNA-AL390243.1 in predicting nodal metastasis was 0.727. *p < 0.05; ***p < 0.0005; ns, no significance.

As these three lncRNA might be nodal efficacy biomarkers after NAT of HER2+ breast cancer, we proposed a hypothesis that these lncRNA might predict nodal metastasis of breast cancer. Next, we selected 48 pairs of breast cancer tissues and para-cancer tissues from patients who did not receive NAT. The expression of lncRNA-AC009975.1, POTEH-AS1, and lncRNA-AL390243.1 were analyzed in the cohort. The lncRNA-AL390243.1, POTEH-AS1, and lncRNA-AC009975.1 were significantly elevated in cancer tissues compared to the para-cancer tissues (**Figure 6A**). The *p* values were 0.0003, 0.0009, and 0.0241, respectively.

We then evaluated the nodal status in cancer tissues. In these 48 cancer tissues, we found that the expression of lncRNA-AL390243.1 was significantly higher in the nodal positive subgroup than in the nodal negative subgroup (*p*=0.0271), whereas POTEH-AS1 and lncRNA-AC009975.1 showed no significant difference among nodal status (all *p >*0.05, **Figure 6B**). The AUC value of lncRNA-AL390243.1 in predicting nodal metastasis was 0.727 (95%CI: 0.554-0.859, *p*=0.0153, **Figure 6C**).

## Discussion

In the current study, the expression of lncRNA-AL390243.1, POTEH-AS1, and lncRNA-AC009975.1 was statistically increased in core biopsy tissues from the non-apCR group compared to those from the apCR group, possessing rather high diagnostic efficiency. The AUC value was 0.789 with a sensitivity of 80.6%, and a specificity of 61.0%. More importantly, according to multivariate analysis, the expression of lncRNA-AL390243.1, nodal stage, and tumor type were independent predictors of apCR after NAT, which also possessed a high diagnostic efficiency with 0.859 (95%CI: 0.790-0.929). The nomogram may be especially helpful in predicting which patients might benefit from NAT concerning nodal response, allowing a more individualized assessment of nodal conversion probability. The expression of lncRNA-AL390243.1 was significantly higher in nodal positive breast cancer than in nodal negative cancer, suggesting the expression of lncRNA-AL390243.1 might be a novel biomarker for nodal metastasis diagnostics. In addition, the high expression of lncRNA-AL390243.1 was associated with poor DFS.

The benefits of NAT for breast cancers have been well described by others (16, 17). Furthermore, the achievement of pathological complete response (pCR) is a surrogate marker of improved oncologic outcomes, especially in HER2+ breast cancer (18–20). The use of effective chemotherapy as well as targeted therapies such as trastuzumab and (or) pertuzumab for HER2+ disease in the neoadjuvant setting have led to an increase in the rate of pCR after NAT ranging from 30 to 63% depending on the study population. However, the bpCR was not completely consistent with apCR. As this difference was most significant in the HER2+ subtype, we aim to explore whether there are differential lncRNAs in primary tumors that could predict apCR after NAT.

Previous studies have demonstrated the potential value of lncRNAs as efficacy prediction biomarkers for breast cancer (11–13). LncRNA H19 could promote drug resistance in HR+ breast cancer cells through inhibiting BIK and NOXA expression (21). Liang et al. (22) found that lncRNA PRLB could act as an oncogene by affecting the miR-4766-5p/SIRT pathway, and significantly increase breast cancer proliferation and chemoresistance. Zhang et al. (23) found that the expression level of lncRNA ITGA9-AS1 in the non-pCR group was much lower than that in the pCR group (AUC value=0.800). Yuan et al. (24) observed that lncRNA ATB could promote trastuzumab resistance by competitively biding miR-200c, upregulating E-box-binding protein 1 and zinc finger protein 217, inducing epithelial-mesenchymal transition and invasion. In addition, Shi et al. (25) also found that the high expression of lncRNA ATB was correlated with trastuzumab resistance of breast cancer patients. Li et al. (26) screened a microarray of lncRNA involved in the trastuzumab-resistant SKBR-3/Tr cells. The expression of lncRNA GAS5 was decreased in SKBR-3/Tr cells. Further research showed that lncRNA GAS5 suppresses cancer proliferation by acting as a molecular sponge for miRNA-21, leading to the de-repression of phosphatase and tension homologs. Moreover, mTOR activation associated with reduced lncRNA GAS5 expression was required to suppress PTEN. This work identified lncRNA GAS5 as a novel prognostic marker and candidate drug target for HER2+ breast cancer.

However, most studies focused on the efficacy prediction of breast primary tumors. The strength of our study was that, to the best of our knowledge, it is the first to explore whether the lncRNA that come from breast primary tumors have nodal efficacy prediction value. The current study demonstrated that lncRNA-AL390243.1, POTEH-AS1, and lncRNA-AC009975.1 were increased in the non-apCR group significantly and stably. We believed that these three lncRNAs could play a crucial role in the nodal efficacy prediction of breast cancer, but the underlying molecular mechanisms require further research.

For patients with initial cN_+_ and ycN_0_ disease after NAT, the feasibility of SLNB has been confirmed by NCCN guidelines (category 2B) and St. Gallen international expert consensus (5, 6, 8). However, the overall FNR of patients undergoing SLNB after NAT is still high. On the one hand, improving the technology of SLNB could help to reduce FNR. On the other, the sensitivity of selection could be improved by optimizing patient selection. If patients have better therapy response and a higher apCR rate, they might have more chances of accepting SLNB while avoiding ALND after NAT (9). In other words, individualized surgical treatment could be chosen according to therapy response after NAT. The transformation of the axillary surgical downstage management after NAT, on the one hand, is based on the efficacy of systemic treatment for local-regional control. On the other hand, it is based on tumor burden (27). However, in clinical practice, we need to understand the complementary relationship between clinical indicators and genomics. Considering the differences between clinical research results and real-world clinical treatment, we need to be cautious when making clinical decisions.

There were several limitations to this study. The most important was the retrospective study design involving a limited number of patients who attended a single institution, which increased the selection bias. Second, were unable to take some factors associated with apCR into account. The clinical application of genomics still needs to be validated using a large, independent, prospective cohort. At the same time, the underlying molecular mechanisms of lncRNA require further study.

In conclusion, our study identified that lncRNA-AL390243.1, POTEH-AS1, and lncRNA-AC009975.1 were upregulated in non-apCR breast cancer tissues. These three lncRNAs might have the potential to be used as predictive biomarkers of nodal efficacy of HER2+ breast cancer but the underlying molecular mechanisms require further study.

## Data Availability Statement

The raw data supporting the conclusions of this article will be made available by the authors, without undue reservation.

## Ethics Statement

Ethical review and approval was not required for the study on human participants in accordance with the local legislation and institutional requirements. The patients/participants provided their written informed consent to participate in this study.

## Author Contributions

All authors made a substantial contribution to the conception or design of the work, including the acquisition, analysis, and interpretation of data. ZB, YZ, and X-GS participated in drafting the work. PC, P-FQ, and LX revised it critically for important intellectual content. Y-SW and X-RS approved the final completed version of this paper and assume accountability for all aspects of the work. All authors contributed to the article and approved the submitted version.

## Funding

This work was funded by the National Natural Science Foundation of China (81672638, 81672104), the Shandong Provincial Key Research and Development Program (2017CXGC1207, 2019GSF108179, 2019GSF108104), and the Shandong Cancer Hospital and Institute Clinical Training Program (20206108).

## Conflict of Interest

The authors declare that the research was conducted in the absence of any commercial or financial relationships that could be construed as a potential conflict of interest.

## Publisher’s Note

All claims expressed in this article are solely those of the authors and do not necessarily represent those of their affiliated organizations, or those of the publisher, the editors and the reviewers. Any product that may be evaluated in this article, or claim that may be made by its manufacturer, is not guaranteed or endorsed by the publisher.
